# Seasonality in swimming and cycling: Exploring a limitation of accelerometer based studies

**DOI:** 10.1016/j.pmedr.2017.04.006

**Published:** 2017-04-29

**Authors:** Flo Harrison, Andrew J. Atkin, Esther M.F. van Sluijs, Andy P. Jones

**Affiliations:** a Norwich Medical School and UKCRC Centre for Diet and Activity Research (CEDAR), University of East Anglia, Norwich NR4 7TJ, United Kingdom; b MRC Epidemiology Unit and UKCRC Centre for Diet and Activity Research (CEDAR), University of Cambridge School of Clinical Medicine, Cambridge CB2 0QQ, United Kingdom

**Keywords:** Physical activity, Seasonality, Children, Millennium Cohort study

## Abstract

Accelerometer-based studies of children's physical activity have reported seasonal patterns in activity levels. However, the inability of many accelerometers to detect activity while the wearer is swimming or cycling may introduce a bias to the estimation of seasonality if participation in these activities are themselves seasonally patterned. We explore seasonal patterns in children's swimming and cycling among a sample of 7–8 year olds (*N* = 591) participating in the Millennium Cohort Study, UK. Participating children wore an accelerometer for one week on up to five occasions over the year and their parents completed a diary recording daily minutes spent swimming and cycling. Both swimming and cycling participation showed seasonal patterns, with 2.7 (SE 0.8) more minutes swimming and 5.7 (0.7) more minutes cycling performed in summer compared to winter. Adding swimming and cycling time to accelerometer-determined MVPA increased the summer-winter difference in MVPA from 16.6 (1.6) to 24.9 min. The seasonal trend in swimming and cycling appears to follow the same pattern as accelerometer-measured MVPA. Studies relying solely on accelerometers may therefore underestimate seasonal differences in children's activity.

## Highlights

• Children's physical activity is higher in spring/summer relative to autumn/winter.
• Accelerometers cannot detect activity while the wearer is swimming or cycling.
• Parent-reported time spent swimming and cycling also varies seasonally.
• Accelerometer-based studies may underestimate seasonal differences in activity.

## List of abbreviations

**Table:** 

cpm	counts per minute
MCS	Millennium Cohort Study
MET	metabolic equivalent
MVPA	moderate to vigorous physical activity
SES	socio-economic status

## 1 Introduction

Several studies have reported a seasonal pattern in young people's objectively measured physical activity, with higher activity levels recorded in the spring and summer than autumn and winter (Loucaides et al., 2004, Riddoch et al., 2007, Cleland et al., 2008, Gracia-Marco et al., 2013, Hjorth et al., 2013, Atkin et al., 2016). An often cited limitation of such work (Loucaides et al., 2004, Rich et al., 2012, Hjorth et al., 2013, Atkin et al., 2016), however, is the inability of devices such as accelerometers and pedometers to record activity while participants are swimming and cycling; they must be removed during aquatic activities and do not detect the full scale of movement when the wearer is cycling (Chen and Bassett, 2005). This may introduce bias into the findings of these studies if these non-recorded activities themselves show seasonal variation. To examine the magnitude of this problem, we describe seasonal variation in parent-reported time spent swimming and cycling in UK children aged 7–8 years from the Millennium Cohort Study.

## 2 Methods and participants

The Millennium Cohort Study (MCS) recruited at birth a sample of 18,818 children from across the UK between September 2000 and January 2002. As part of the fourth follow-up survey at 7 years (MCS4), 1289 participants were invited to participate in a seasonal physical activity study and asked to wear an ActiGraph GT1M accelerometer (ActiGraph, Pensacola, FL) five times; in winter 2008/09, spring, summer and autumn of 2009 and winter 2009/10 (University of London. Institute of Education. Centre for Longitudinal Studies and University College London., 2013). At each measurement occasion, participants were asked to wear the device during waking hours for seven consecutive days, removing it during aquatic activities.

Accelerometers were set to record in 15-s epochs. Periods of ≥ 20 min of consecutive zero counts and count values of ≥ 11,715 counts per minute (cpm) were removed as non-wear time and unfeasibly high recordings respectively. A minimum of 2 days of valid data (> 10 h wear time) at any assessment wave was required for inclusion in the analysis. Two days of measurements has previously been found to be the minimum required for a reliable estimate of children's activity in the MCS (Rich et al., 2013). Moderate-to-vigorous physical activity (MVPA) was defined as that > 2241 cpm (Pulsford et al., 2011), which equates to walking at approximately 4 km per hour in children (Trost et al., 1998).

Participants' parents were asked to complete a timesheet over each week of accelerometry, in which they recorded how many minutes their child spent swimming and cycling each day. We included measurement occasions in these analyses if a numeric value (≥ 0) was recorded both for minutes spent swimming and cycling on at least two of the seven days, and we coded any missing values to zero; doing so was found to produce estimates of mean time spent swimming and cycling that more closely resembled mean time spent in the activities for those participants with no missing values (see Supplementary Fig. 1).

We calculated mean daily minutes spent swimming and cycling, and mean daily minutes of MVPA as recorded by accelerometry for each measurement occasion. Seasons were defined based on solstices and equinoxes in line with previous work with this sample (Atkin et al., 2016). Measurement occasions were assigned to seasons based on the date of the first day of accelerometry.

Swimming and cycling are deemed to have metabolic equivalent (MET) values > 3, and so would contribute to total MVPA (Ridley et al., 2008). In order to assess the impact of not including swimming and cycling in the assessment of seasonal patterns in MVPA we summed minutes of swimming, cycling and accelerometer-measured MVPA. Multilevel regression models, allowing for the clustering of measurement occasions within children, were run to explore seasonal patterns in four outcomes: daily minutes swimming, cycling, accelerometer-determined MVPA, and MVPA plus swimming and cycling. As the mix of study participants showed some variation by season, models were adjusted for sex, age, weight status (from objectively-measured height and weight at MCS4) and accelerometer wear time (where appropriate).

## 3 Results

Of the 1289 children invited, 705 (55%) consented to taking part in the seasonal study. Of these, 114 did not provide valid records of MVPA, or swimming and cycling on at least one occasion, leaving a final sample of 591 (46%). Table 1 shows the characteristics of those included. The participants included in these analyses were not significantly different from the main MCS4 sample in terms of age and sex, but were less likely to be overweight or obese; 20% of the main sample were overweight or obese compared to 15% in the analytical sample (*p* = 0.007).

**Table 1:** Participant characteristics.

		N(%) or mean, SD
Total		591
Sex	Female	302 (51.1%)
	Male	289 (48.9%)
Weight status	Not overweight	499 (84.4%)
	Overweight	66 (11.2%)
	Obese	26 (4.4%)
Age (years)		7.25, 0.23
Mean daily minutes MVPA	Winter	51.63, 18.24
	Spring	69.55, 25.87
	Summer	67.06, 23.28
	Autumn	57.63, 19.62
Mean daily accelerometer wear time	Winter	731.33, 51.34
	Spring	732.08, 51.43
	Summer	725.08, 52.63
	Autumn	735.97, 56.39

**Fig. 1:**
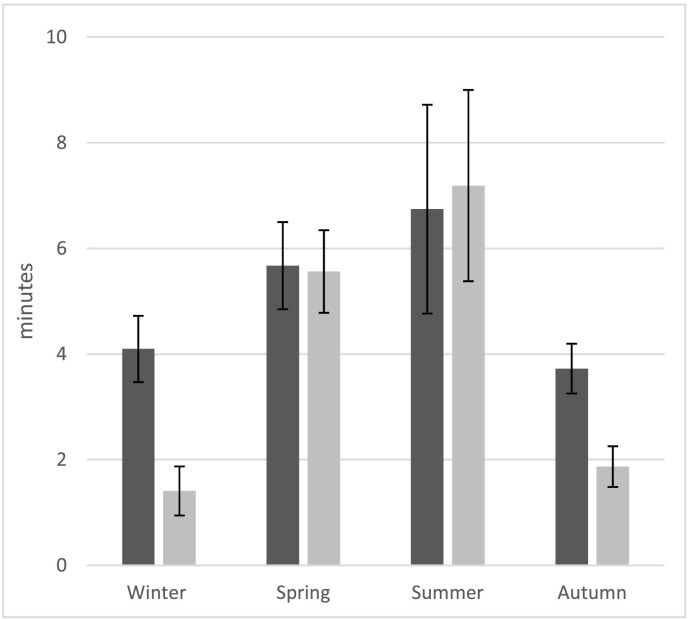
Mean (95% CI) daily reported minutes spent swimming (■) and cycling (■) by season.

**Fig. 2:**
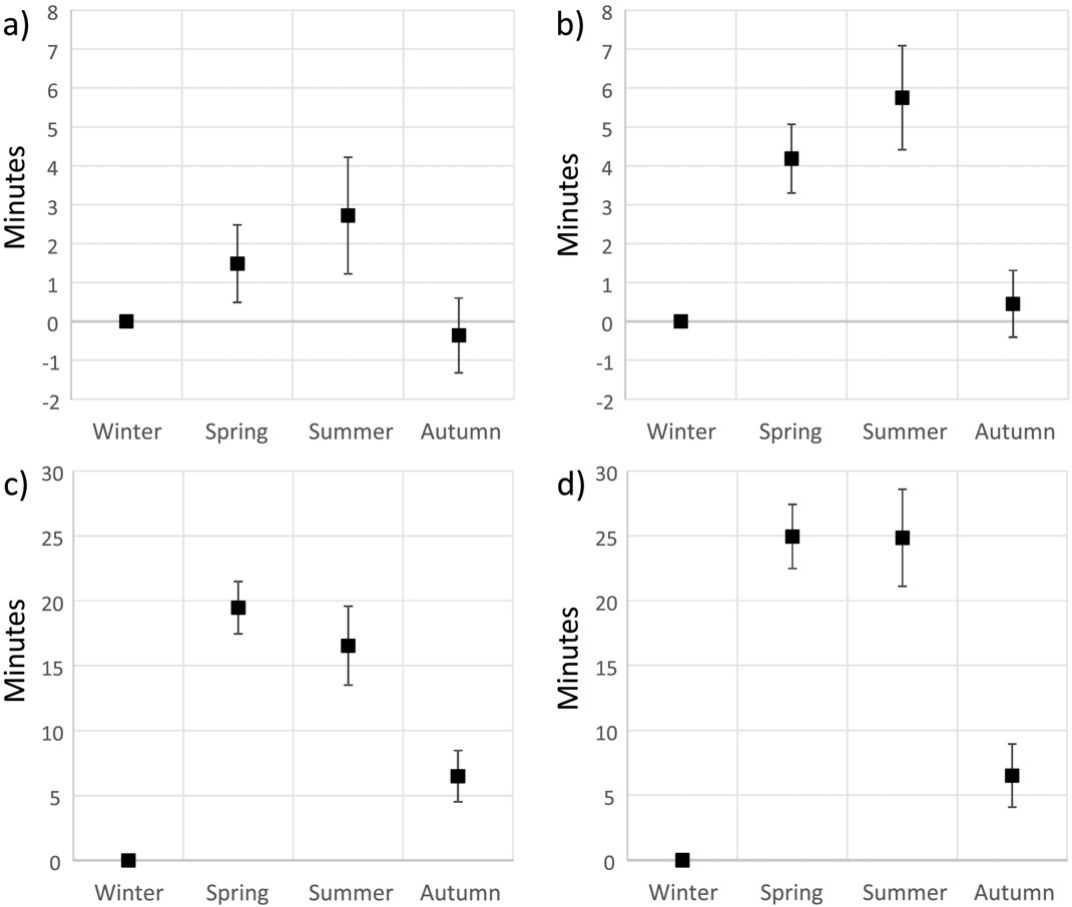
Mean difference (with 95% CI) in daily time spent: a) self-reported-measured MVPA + reported time spent swimming and cycling). All adjusted for sex, age, weight status, and accelerometer wear-time (figure c and d), in multilevel models adjusted for clustering of measurements within children.

Fig. 1 shows reported mean daily minutes spent swimming and cycling in the four seasons. Both activities show a clear seasonal pattern with greater participation in the spring and summer compared to autumn and winter. Fig. 2 shows the regression coefficients for the four outcomes, indicating the average difference in daily time spent in each activity by season relative to winter. Children spent on average 2.7 (SE 0.8) minutes per day more swimming in the summer compared to the winter (Fig. 2a), and 5.7 (0.7) minutes per day more cycling (Fig. 2b). When time spent swimming and cycling was added to accelerometer measured MVPA, the seasonal pattern seen in MVPA is accentuated; the differences between summer and winter increasing from 16.6 (1.6) minutes (Fig. 2c) to 24.9 (1.9) minutes (Fig. 2d). This equates to a mean daily estimated MVPA of 60.2 (1.3) vs 65.5 (1.5) in the winter and 76.8 (1.7) vs 90.4 (2.0) in the summer.

## 4 Discussion

Parent-reports of 7–8 year olds' time swimming and cycling show seasonal patterns, with more time spent in both activities during spring and summer compared to autumn and winter. These trends follow the same pattern as accelerometer-measured MVPA of which participants did an average of 20 min more in the spring compared to the winter. Given that shorter day length and inclement weather are usually cited as the reasons for reduced activity in the winter (Rich et al., 2012), it is particularly noteworthy that swimming, a predominantly indoor activity in the UK, shows a similar seasonal trend.

As the MVPA accrued while swimming and cycling is not accurately captured by accelerometry, the seasonal patterns in participation in these activities shown here have the potential to bias assessment of seasonal patterns in activity. Our results suggest that examinations of seasonal variations in physical activity that rely on accelerometry may in fact be under-estimating the magnitude of seasonal variation in activity. However, while the addition of swimming and cycling minutes to overall MVPA heightens the seasonal pattern observed, it does not alter the general trend of overall greater activity levels in the spring and summer compared to other times.

If we are to support children being more active throughout the year, it is important to consider what types of activities they might be most inclined to do in the winter months. These analyses suggest that there are seasonal differences in the time spent in specific activity types, and further investigation of seasonality in self-reported activity, coupled with qualitative work on activity choices, may provide useful information.

Some limitations must be acknowledged. Time spent swimming and cycling was reported by participant's parents and the validity of these estimates is unknown. A substantial proportion (40%) of study days were missing information on time spent swimming and cycling, which we coded to zero. We tested this assumption by re-running our analyses with only those with no missing data, and by assuming missing days are missing. The former analysis produced almost identical results, while the latter increased the estimated time spent swimming and cycling, but did not alter seasonal differences in participation. Similarly, tightening the inclusion criteria to ≥ 4 days of non-missing swimming and cycling time did not significantly alter our results. We have added reported time spent swimming and cycling to accelerometer-measured MVPA to give an indication as to the potential impact of the non-recording of these activities. However, we do not know if all this time would actually meet the threshold for MVPA, and may actually have constituted light activity or even sedentary time. The MCS seasonal study included measurements from across the year including school and holiday time. Little is known about any interaction between school vs holiday time and seasonality in physical activity, although an interaction has been found between season and weekend/weekdays, whereby greater seasonal variation in activity is observed for weekend days compared to during the week (Atkin et al., 2016). Unfortunately, the information on week type (term-time/holiday time) that is available in MCS is not complete. When completing the swimming and cycling log, parents were asked to report whether the week had been a school week, a holiday week or both. This question was not asked during the first round of data collection (Winter 08/09), and is missing for 34% of our sample. The information that is available shows that the distribution of school/holiday weeks does vary by season. Around 45% of spring measurements were in holiday weeks compared to 34% in summer, 17% in Autumn and 1% in Winter. In sensitivity analyses including week type as a covariate did not substantially alter our findings (results not presented). This is an area that may warrant further investigation in future studies. Finally, our sample was less likely to be overweight than the wider MCS sample, possibly limiting the generalisability of these results.

To conclude, we found evidence of a seasonal pattern in 7–8 year olds' participation in swimming and cycling. These patterns follow the same trend as that seen in accelerometer-measured MVPA, with higher participation in spring and summer compared to autumn and winter. Studies relying solely on accelerometers may therefore underestimate seasonal differences in activity, and estimates of potential health gains in season-based interventions, such as a change to daylight savings (Goodman et al., 2014), may therefore also be conservative.

The following is the supplementary data related to this article.

Supplementary Fig. 1Comparison of mean minutes spent (A) swimming and (B) cycling per season using different approaches to missing data handling.  only those participants with no missing days (*N* = 313),  all participants with at least 2 days non-missing (*N* = 591) with missing values treated as missing (i.e. if numeric values were recorded on two days, these values were summed and divided by 2), and  all participants with at least 2 days non-missing (*N* = 591) with missing values coded to 0 (i.e. if numeric values were recorded on two days, these values were summed and divided by 7).Supplementary Fig. 1

## Database linking

The datasets supporting the conclusions of this article are available in the UK Data Archive, SN: 7238, http://dx.doi.org/10.5255/UKDA-SN-7238-1.

## Competing interests

The authors declare that they have no competing interests.

## Funding

The fourth sweep of the MCS was funded by grants to Professor Heather Joshi, former director of the study, from the Economic and Social Research Council and a consortium of government funders. The current director is Professor Lucinda Platt. The authors acknowledge the Centre for Longitudinal Studies, Institute of Education, for the use of these data; the UK Data Service for making them available; and the MRC Centre of Epidemiology for Child Health (grant reference G0400546), Institute of Child Health, University College London, for creating the accelerometer data resource, which was funded by the Wellcome Trust (grant reference 084686/Z/08/A). The institutions and funders acknowledged bear no responsibility for the analysis or interpretation of these data.

The work of Andrew J Atkin, Flo Harrison, and Esther M F van Sluijs was supported, wholly or in part, by the Centre for Diet and Activity Research (CEDAR), a UKCRC Public Health Research Centre of Excellence (RES-590-28-0002). Funding from the British Heart Foundation, Department of Health, Economic and Social Research Council, Medical Research Council, and the Wellcome Trust, under the auspices of the UK Clinical Research Collaboration, is gratefully acknowledged. The work of Esther MF van Sluijs was supported by the Medical Research Council (MC_UU_12015/7).

## Transparency document

Transparency document. Image 1
