# Supplementary material for: Seasonality in swimming and cycling: Exploring a limitation of accelerometer based studies
Source: Prev Med Rep. 2017 Apr 29;7:16–9. doi: 10.1016/j.pmedr.2017.04.006 (PMC5447377; doi:10.1016/j.pmedr.2017.04.006)
Supplement: Supplementary Fig. 1 — Comparison of mean minutes spent (A) swimming and (B) cycling per season using different approaches to missing data handling. Image 2 only those participants with no missing days (N = 313), Image 3 all participants with at least 2 days non-missing (N = 591) with missing values treated as missing (i.e. if numeric values were recorded on two days, these values were summed and divided by 2), and Image 4 all participants with at least 2 days non-missing (N = 591) with missing values coded to 0 (i.e. if numeric values were recorded on two days, these values were summed and divided by 7). [file mmc1.pdf]

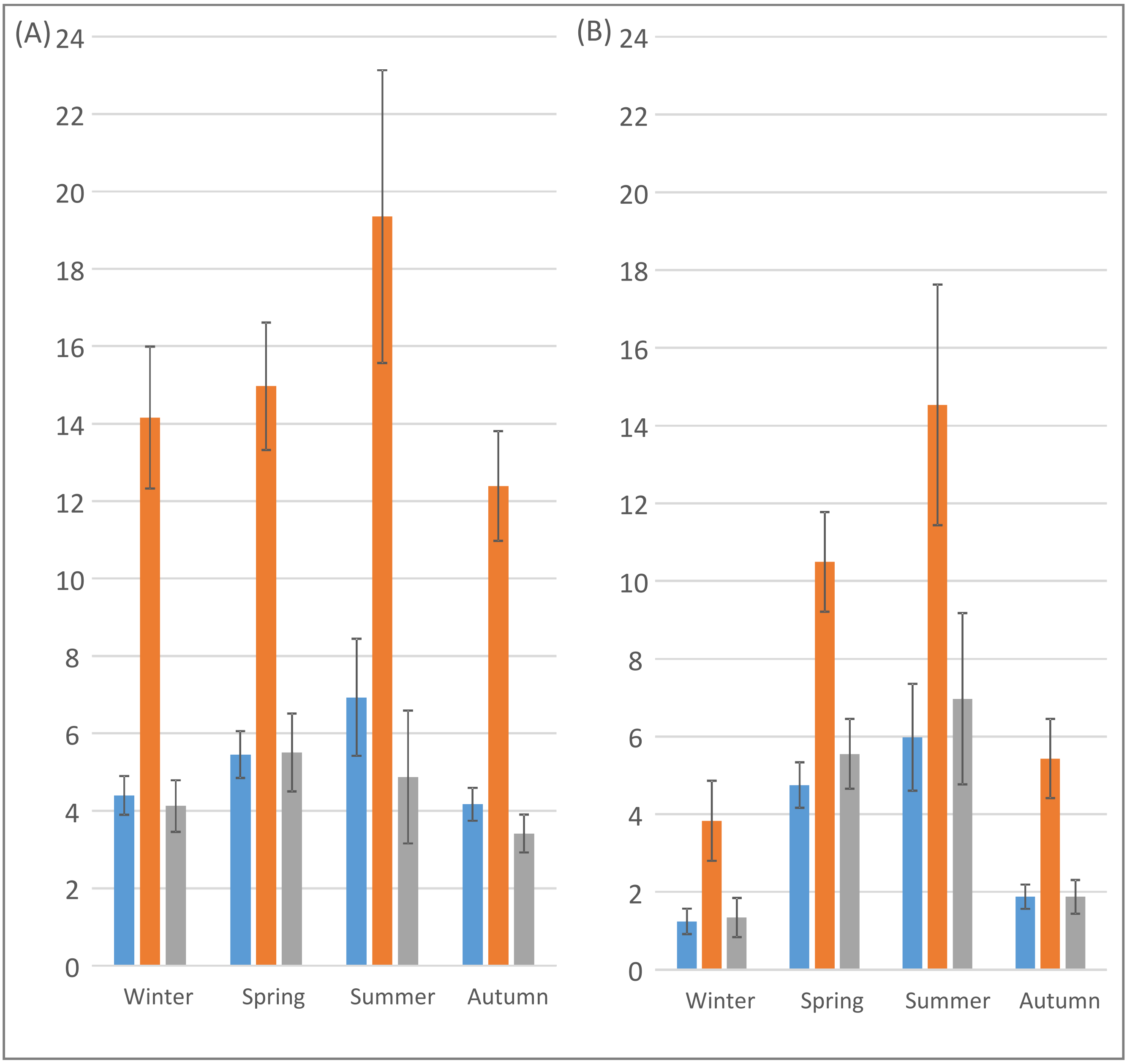

Supplementary Figure 1. Comparison of mean minutes spent (A) swimming and (B) cycling per season using different approaches to missing data handling. ■ only those participants with no missing days (N=313), ■ all participants with at least 2 days non-missing (N=591) with missing values treated as missing (i.e. if numeric values were recorded on two days, these values were summed and divided by 2), and ■ all participants with at least 2 days non-missing (N=591) with missing values coded to 0 (i.e. if numeric values were recorded on two days, these values were summed and divided by 7).
